# Feature Selection Using Correlation Analysis and Principal Component Analysis for Accurate Breast Cancer Diagnosis

**DOI:** 10.3390/jimaging7110225

**Published:** 2021-10-26

**Authors:** Sara Ibrahim, Saima Nazir, Sergio A. Velastin

**Affiliations:** 1 Department of Computer Science, Capital University of Science and Technology, Islamabad 45730, Pakistan; sara.ibrahim@cust.edu.pk; 2 Department of Software Engineering, National University of Modern Languages, Rawalpindi 46000, Pakistan; 3 Applied Artificial Intelligence Research Group, Department of Computer Science and Engineering, University Carlos III de Madrid, 28270 Madrid, Spain; sergio.velastin@ieee.org; 4 School of Electronic Engineering and Computer Science, Queen Mary University of London, London E1 4NS, UK

**Keywords:** breast cancer diagnosis, Wisconsin Breast Cancer Dataset, feature selection, dimensionality reduction, principal component analysis, ensemble method

## Abstract

Breast cancer is one of the leading causes of death among women, more so than all other cancers. The accurate diagnosis of breast cancer is very difficult due to the complexity of the disease, changing treatment procedures and different patient population samples. Diagnostic techniques with better performance are very important for personalized care and treatment and to reduce and control the recurrence of cancer. The main objective of this research was to select feature selection techniques using correlation analysis and variance of input features before passing these significant features to a classification method. We used an ensemble method to improve the classification of breast cancer. The proposed approach was evaluated using the public WBCD dataset (Wisconsin Breast Cancer Dataset). Correlation analysis and principal component analysis were used for dimensionality reduction. Performance was evaluated for well-known machine learning classifiers, and the best seven classifiers were chosen for the next step. Hyper-parameter tuning was performed to improve the performances of the classifiers. The best performing classification algorithms were combined with two different voting techniques. Hard voting predicts the class that gets the majority vote, whereas soft voting predicts the class based on highest probability. The proposed approach performed better than state-of-the-art work, achieving an accuracy of 98.24%, high precision (99.29%) and a recall value of 95.89%.

## 1. Introduction

Breast cancer is one of the leading causes of death among women [1]. Although cancer is largely preventable in its primary stages, there are still many women who are diagnosed with cancer at a late stage. Better performing diagnosis techniques are very important in personalized care and treatment, and the use of such techniques can also help to control and reduce the recurrence of cancer. In the medical field, clinicians generally use data from various sources, such as medical records, laboratory tests, and studies related to the disease for accurate diagnosis and prediction of breast cancer. The use of artificial intelligence (AI) techniques in the medical field is also increasing to automate disease diagnosis and to get better results in terms of performance.

Breast cancer occurs in breast cells of the fatty tissues or the fibrous connective tissues within the breast. Breast cancer is a type of tumor that tends to become gradually worse and that grows fast, which leads to death. Breast cancer is more common among females, but it can also occur among males, although rarely. Various factors, such as age and family history, can also contribute to breast cancer risk. Two main types of breast tumors can be identified.

Benign: If the cells are not cancerous, the tumor is benign (not dangerous to health). It will not invade nearby tissues or spread to other areas of the body (metastasize). A benign tumor is not worrisome unless it is pressing on nearby tissues, nerves, or blood vessels and causing damage.

Malignant: This means that the tumor is made of cancerous cells and it can invade nearby tissues and thus be potentially hazardous. Some cancer cells can move into the bloodstream or lymph nodes, where they can spread to other tissues within the body, which is known as metastasis. This is a tumor that is more dangerous and causes death. The main types or forms of breast cancer include:

1. Ductal carcinoma in situ (DCIS): It is the earliest stage of breast cancer and can be diagnosed and is curable. The vast majority of women diagnosed with it get cured. Although it is non-invasive, it might lead to invasive cancer.
2. Invasive ductal carcinoma (IDC): It begins in the milk duct and can spread to the surrounding breast tissues. It is the most common type of breast cancer.
3. Invasive lobular carcinoma (ILC): It starts in a lobule of the breast. It can spread fast to the lymph nodes and other areas of the body.

Approximately one million females are diagnosed with breast cancer approximately every year worldwide. As many as 81% of females with early-stage breast cancer survive for five years. However, only 35% of females with late or advanced-stage breast cancer survive for five years. The work proposed here highlights the significance of the use of the best performing machine learning classifiers with ensembles techniques for accurate diagnosis of breast cancer. The objective of the proposed research was to implement a feature reduction algorithm which can find a subset of features that can guarantee a highly accurate breast cancer classification as either benign or malignant. Principal component analysis (PCA) was used for dimensionality reduction and hyper-parameter tuning was performed to gain performance. We also compared different state-of-the-art machine learning classification algorithms. We used the publicly available Wisconsin Breast Cancer Dataset (WBCD) [2], and its features were computed from a digitized image of a fine needle aspirate (FNA) of a breast mass. They describe the characteristics of the cell nuclei present in the image. We evaluated the performances of logistic regression, support vector machine, k-nearest neighbors, stochastic gradient descent learning, naïve Bayes, random forest, and decision tree. These seven classifiers were used for further processing and ensembled with voting techniques that included hard voting and soft voting.

The rest of the paper is structured as follows: Section 2 describes related work and different state-of-the-art approaches used for breast cancer diagnosis. Section 3 describes the proposed framework and related details of the proposed work. Section 4 deals with experimentation and discussion. Section 5 presents a comparison with the state-of-the-art. Section 6 ends the paper with conclusions and proposed future work.

## 2. Literature Review

Many studies have used artificial intelligence (AI) techniques for breast cancer diagnosis to enhance the accuracy of classification and its speed. Here, we have reviewed some relevant work dealing with breast cancer diagnosis that has used machine and deep learning approaches.

Nguyen et al. [3] used the WBCD Dataset to evaluate the performance of supervised and unsupervised breast cancer classification models. Scaling and principal component analysis were used for the selection of features, and they split the data into a 70:30 ratio for training and testing. They argued that the ensemble voting method is suitable as a prediction model for predicting breast cancer. After feature selection techniques, various models were tested and trained on the data. Among all the models used for the prediction, they stated that only four models, i.e., ensemble-voting classifier, logistic regression, support vector machine, and adaboost, provided approximately 90% accuracy. They reported the performance of the proposed model using accuracy, recall tests, ROC-AUC (receiver operating characteristic curve- area under the curve), F1-measure, and computational time.

To compare the models, the data from the Iranian Center for Breast Cancer dataset were analyzed to explore risk factors in breast cancer prediction. Ahmed et al. [4] used decision trees (DTs), artificial neural networks (ANNs), and support-vector machines (SVMs). The results show that SVM outperformed both the decision tree and the MLP (multilayer perceptron) in all the parameters of sensitivity, specificity, and accuracy. There are some limitations to their study, as many cases were lost in the follow-up and there were records with missing values that were omitted. Apart from missing data, some important variables such as S-phase fraction and DNA index were not included in the study because of their unavailability, which may have decreased the performance of the models.

Omondiagbe et al. [5] discussed the classification of different types of breast cancer (benign and malignant) in the Wisconsin Diagnostic datasets using support vector machine (SVM), artificial neural network (ANN), and naive Bayes approaches. Their main goal was to propose the most suitable approach by integrating machine learning techniques with different feature selection/feature extraction methods. They proposed a hybrid approach for breast cancer diagnosis by reducing the high dimensionality of features using LDA (linear discriminant analysis) and then applying the new reduced feature dataset to a support vector machine. Their approach showed 98.82% accuracy, 98.41% sensitivity, 99.07% specificity, and 0.9994 area under the receiver operating characteristic curve (AUROC).

Yesuf et al. [6] used the CFS (correlation based feature selection) technique for feature selection in which a 0.7 correlation filter value was set and features with means above 0.7 were omitted from the training dataset. Another technique used for feature selection was recursive feature elimination (RFE) [7], which used the wrapper approach. In that approach, all the feature subsets were rated on the basis of accuracy score, and subsets were selected which had features having top ranking scores. Their research was on the basis of a technique that used PCA (principal component analysis) on neural networks. They used PCA and LDA for feature extraction and CFS and RFE for feature selection.

Jamal et al. [8] worked on two machine learning algorithms, a support vector machine (SVM) and extreme gradient boosting, and compared their performances. For classification they reduced the number of data attributes by extracting the features with the help of principal component analysis (PCA) and clustering with k-means. They reported the performances of four models using accuracy, sensitivity, and specificity from confusion matrices. Their results indicated that k-means was the best method, which was not generally used for dimensionality reduction, but can perform well compared to PCA. Four algorithms were employed—namely, PCA, factor analysis, linear discriminant analysis, and multidimensional scaling. The result of simulation on the WBCD showed that maximum accuracy was obtained by the use of PCA and a back-propagation neural network.

Subrata et al. [9] proposed the diagnosis of breast cancer by comparing naïve Bayes (NB), logistic regression (LR), and decision tree (DT) classifiers; the time complexity of each of the classifiers was also measured. It was concluded that the logistic regression classifier was the best classifier with the highest accuracy as compared to the other two classifiers. Kumar et al. [10] worked on the WBCD dataset and evaluated the performance of their proposed work on with adaboost, a decision table, J48, logistic regression, Lazy IBK, Lazy K-star, a multiclass classifier, a multilayer–perceptron, naïve Bayes, J-Rip random forest, and a random tree.

Lucas et al. [11] used Bayesian network and decision tree machine learning classifiers on the WBCD dataset. The Bayesian network gave the best accuracy of 97.80%. Bharat et al. [12] evaluated the performance of their proposed work with three popular machine learning classifiers: naïve Bayes, J48, and RBF networks. The models showed that naïve Bayes obtained the best accuracy of 97.3%, followed by RBF with 96.77%, and J48 came up with 93.41%.

Ravi et al. [13] worked on the Extensible Breast Cancer Prognosis Framework (XBPF) for breast cancer prognosis, which included susceptibility or risk assessment, recurrence, or redevelopment of cancer after the resolution, and survivability. A representative feature for subset selection (RFSS) algorithm was used along with SVM to improve efficiency in prognosis. SVM-RFSS showed a significant performance improvement over state-of-the-art prognosis methods. Chaurasia et al. [14] used three common machine learning classifiers: Bayes’ theorem, a radial basis function network, and decision tree J48. They acquired the UCI dataset (683 instances). They further applied techniques on this dataset such as data selection, preprocessing, and transformation for the development of accurate diagnosis models. The results showed that the naive Bayes performed better, having classification accuracy of 97.36%; and the next two, RBF network and J48, showed 96.77% and 93.41%, respectively.

Haifeng and Won Yoon [15] presented a study on breast cancer diagnosis using different machine learning classifiers. They formulated an effective way to predict breast cancer based on patients’ clinical records. They used four machine learning classifiers: support vector machine (SVM), artificial neural network (ANN), naive Bayes classifier, and adaboost tree. They used two datasets: Wisconsin Diagnostic Breast Cancer and WBCD. In their research work, they also discussed feature space reduction, proposed a hybrid network between various machine learning models and principal component analysis (PCA), and implemented the k-fold cross-validation for the estimation of test errors for each models to select the best method. They also suggested that there were some other models, such as k-means, which can be used for feature space reduction.

Abdollel et al. [16] used relative and absolute area density-based breast cancer measurements. They assessed cancer diagnosis through time of screening mammography and took 392 images from effected cases of breast cancer and 817 images from age matched controls. Multi-variable logistic regression and AUROC (area under the receiver-operating characteristic) were used to assess three risks models. The first model used clinical risk factors, the second model used measures of density-related images, and a third model used clinical risk factors and density-related measurements. They reported that the clinical risk factors model had an AUROC of 0.535, the second model got an AUROC of 0.622, and the third model gave the best result—0.632—outperforming the clinical risk model.

Shravya et al. [17] focused on improving predictive models aimed at high performance in diagnosis of disease outcomes with the help of supervised machine learning methods. They proposed and analyzed the implementations of different machine learning classifiers, logistic regression (LR), support vector machine (SVM), and k nearest neighbors, on the WBCD dataset. SVM performed best with an accuracy of 92.7%.

William et al. [18] focused on naïve Bayes and the J48 decision tree, two machine learning classifiers, to predict breast cancer risks in patients in Nigeria. The J48 decision tree proved to be the most efficient and effective method for predicting breast cancer with the help of highest accuracy level of 94.2% and low error rates as compared to naïve Bayes, having accuracy of 82.6%. Recently, several researchers proposed machine learning (ML) methods for classifying breast abnormality in mammogram images. Assiri et al. [19] proposed an ensemble classifier based on a majority voting mechanism. The performances of different state-of-the-art ML classification algorithms were evaluated for the WBCD dataset. Their classifier achieved an accuracy of 99.42%.

Darzi et al. [20] addressed feature selection for breast cancer diagnosis. They presented a process with a genetic algorithm (GA) and case-based reasoning (CBR). The genetic algorithm was used for searching the problem space to find all of the possible subsets of features, and case-based reasoning was employed to estimate the evaluation result of each subset. The results show that the proposed model performed comparably to the other models on the WBCD dataset. They achieved an accuracy of 97.37%, after feature selection.

When dealing with data that do not have a significant number of training samples, unsupervised machine learning techniques have also proven to be of significant importance in biomedical applications. Marrone et al. [21] have used the 2D fuzzy c-means (FCM) clustering along with geometrical breast anatomy characterization through well defined keypoints. They used FCM to shift the base mask extraction from a simple gray-level-based segmentation to a membership probability. They stated that key point characterization of breast anatomy can be effectively used to weight FCM membership probability, allowing one to accurately separate pectoral muscle from the chest wall. Rundo et al. [22] also used the fuzzy c-means algorithm for the automatic detection and delineation of the necrotic regions within the planned GTV for neuro-radiosurgery therapy.

Most of the published literature has evaluated the performances of classifiers based on accuracy, i.e., a value that is higher when the frequencies of true positives (TPs) and true negatives (TNs) are high compared to those of false positives (FPs) and false negatives (FNs). However, measuring performance in terms of false negatives (recall) and false positives (precision) and F-measures score is equally important, because missing a condition could have serious consequences for patients.

## 3. Methodology

In this paper, an ensemble method is proposed for accurate breast cancer classification, which was made by selecting the appropriate features for processing.

The public UCI breast disease dataset (WBCD) [2] was used as input data. The large size of the dataset and the multiple sources make the data highly useful. WBCD contains 569 instances and 32 attributes. We split the data into a ratio of 70:30 for training and testing. For splitting the dataset, we used the Scmap plot showing the correlaiKit-Learn library in Python—the train-test-split method. Details about the libraries used are mentioned in Appendix A. The training set contained a known output, and the model learned on this data in order to be generalized to other data later on. We used the test dataset (or subset) in order to test our model’s prediction on this subset.

**Figure 1:**
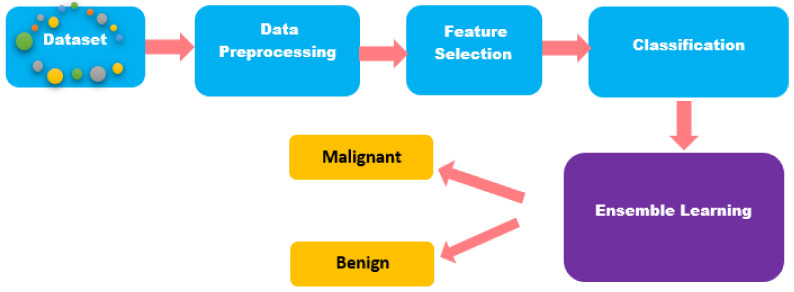
Proposed methodology for accurate breast cancer tumor classification.

The pre-processing of the data was done via data cleaning, data transformation, and normalization. As shown in Figure 1, after pre-processing, we performed feature selection and dimensionality reduction by analyzing the correlation and variance of the input features. Later, the most significant features were used for classification using seven state-of-the-art classification algorithms: logistic regression, support vector machine, k-nearest neighbors, stochastic gradient descent learning, naïve Bayes, random forest, and decision tree. Later, these classifiers were ensembled using voting-based ensembled methods. Hard voting predicts the class that gets the majority vote and soft voting predicts the class based on highest probability. Details about each step of the proposed methodology are given in the sections below.

### 3.1. Data Pre-Processing

Data pre-processing was performed to improve data quality and get a clean dataset which could be used for building the model. Without pre-processing, several challenges will occur—inconsistencies, error, noise, missing values, model over-fitting, etc. To evaluate the impacts of the pre-processing steps on the results of the classification algorithms, breast cancer diagnosis was evaluated separately with and without pre-processing. For pre-processing, we used two feature selection methods and chose the better performing one.

#### 3.1.1. Dimensionality Reduction Using Correlation Analysis

**Figure 2:**
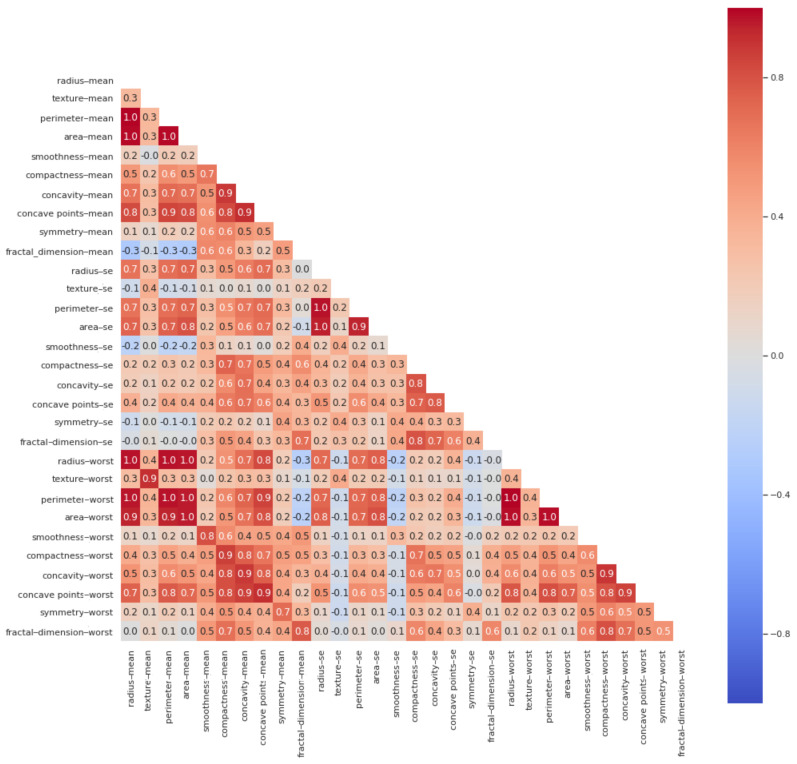
Heat map plot showing the correlations among input features of WBCD dataset.

Dimensionality reduction is a technique to remove features that are less significant for predicting the outcome(s). In this work, dimensionality reduction was performed by analyzing the correlations among the features of input data, dropping features that had high variance. As shown in Figure 2, a heat map was used to analyze the correlations between features of the dataset. A high correlation was observed among “radius-mean”, “parametric-mean”, and “area mean” features, as all these features contain information about the size of breast cancer cells. Therefore, only the “radius-mean” feature was selected to further represent the information about the size of breast cancer cell.

**Figure 3:**
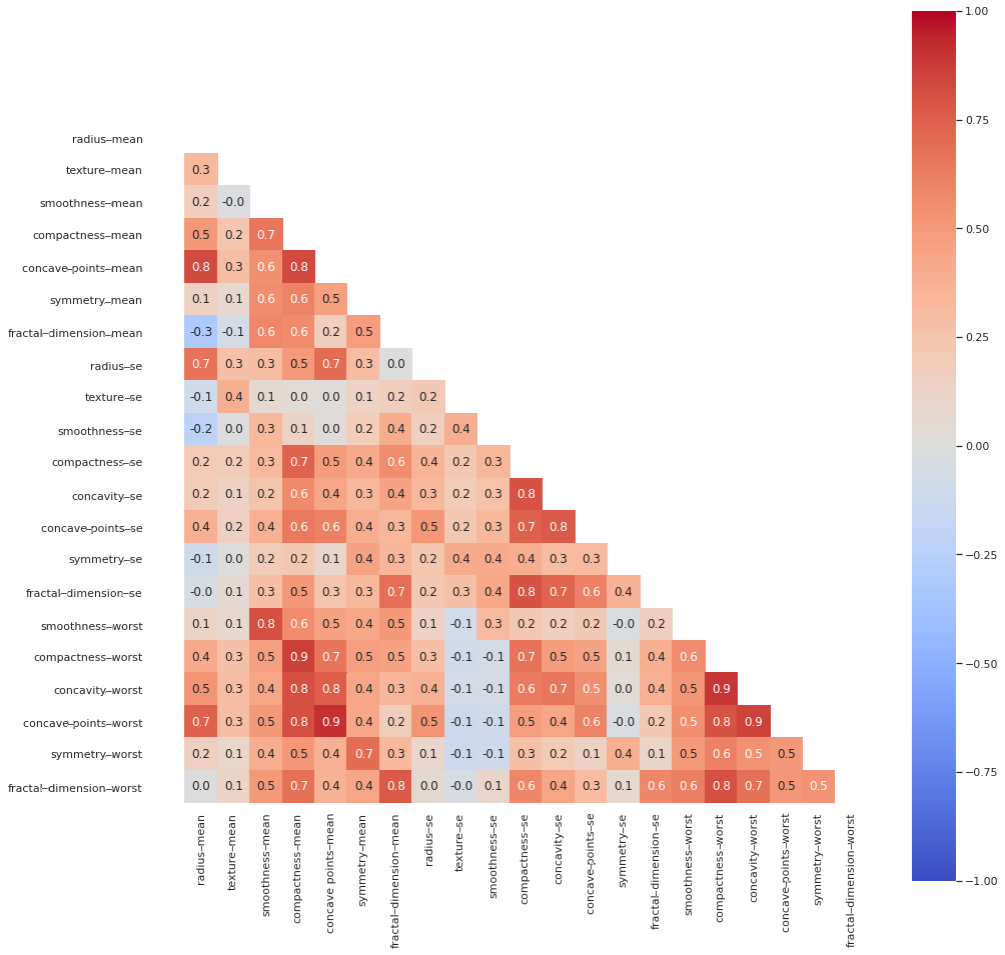
Heat map plot showing the correlations among selected features of WBCD dataset.

High correlations were observed between the features representing the “mean” and “worst” values of different features. For instance, the “radius-mean” feature is highly correlated with the “radius-worst” feature. The feature representing the “worst” value of “radius” was dropped, as it is just a subset of the “mean” value feature. Similarly, high correlations were observed between the features containing information about the shape of breast cancer cell—i.e., compactness, concavity, and concave points. For better breast cancer cell shape representation, we decided to only consider the “compactness-mean” feature for further processing. We dropped a total of nine features: “area-mean, perimeter-mean, radius-worst, area-worst, perimeter-worst, texture-worst, concavity-mean, perimeter-se, area-se.” This way, we had 22 features remaining for further processing. Figure 3 shows the correlations among the selected features.

#### 3.1.2. Dimensionality Reduction Using Principal Component Analysis

The selected features were further analyzed based on their variance. To perform dimensionality reduction based on their variance, we used the well-known principal component analysis (PCA) algorithm. We used the sklearn library, the sklearn.decomposition function, to import PCA (linear dimensionality reduction using singular value decomposition of the data to project it to a lower dimensional space) for feature selection. For PCA we had to ensure that all features were on the same scale; otherwise, the features that have high variance would have affected the outcomes of the PCA. “StandardScaler” was used to standardize features, followed by PCA for dimensionality reduction.

**Figure 4:**
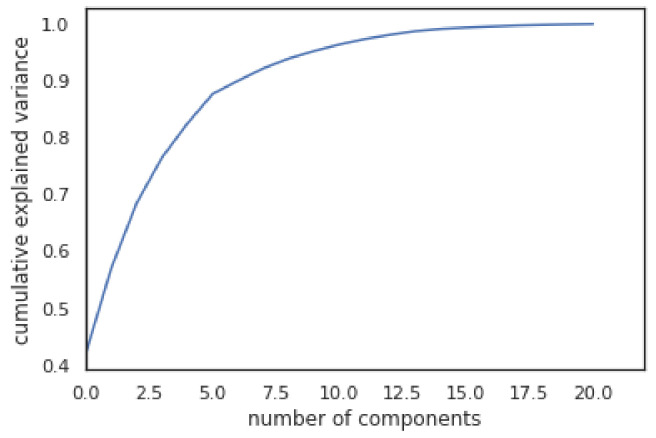
Number of features and their cumulative variance in the WBCD dataset.

Figure 4, shows the variance of different features for the dataset. This graph shows that most of variance can be represented using 10 features only. These 10 features are “radius-mean, texture-mean, compactness-mean, concave points-mean, symmetry-mean, fractal-dimension-mean, smoothness-mean, radius-se, texture-se, and smoothness-se.”

#### 3.1.3. Feature Selection by Using a Wrapper Subset Selection Method

**Figure 5:**
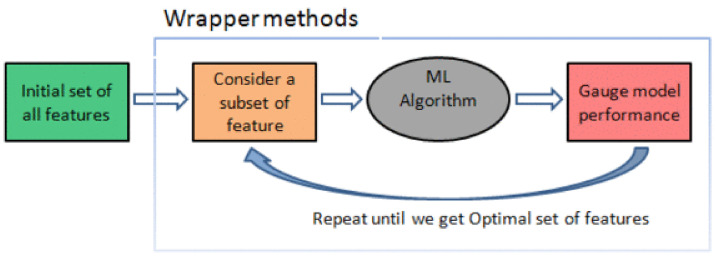
Wrapper method.

We used a wrapper subset selection method for feature selection. Wrapper methods work by evaluating a subset of features using a machine learning algorithm that employs a search strategy to look through the space of possible feature subsets, evaluating each subset based on the quality of the performance of a given algorithm. Wrapper methods generally result in better performance than filter methods because the feature selection process is optimized for the classification algorithm to be used. However, wrapper methods are too expensive for high dimensional data in terms of computational complexity and time, since each feature set considered must be evaluated with the classifier algorithm used. The working of wrapper methods is illustrated in Figure 5,

Summarizing, wrapper methods work in the following way.

- Search for a subset of features: Using a search method, we select a subset of features from the available ones.
- Build a machine learning model: In this step, a chosen ML algorithm is trained on the previously-selected subset of features.
- Evaluate model performance: Finally, the newly-trained ML model is evaluated with a chosen metric.
- Repeat: The whole process starts again with a new subset of features, a new trained ML model. The process stops when the desired condition is met, at which point the subset with the best result in the evaluation phase is chosen.

As a part of first step of feature selection, the search method used was BestFirst, and the chosen machine learning classifier was J48; we set the values of fold to 10, seed to 1, and threshold to −1.0. BestFirst selects the *n* best features for modeling a given dataset, using a greedy algorithm. It starts by creating *N* models, each of them using only one of the *N* features of the dataset as input. The feature that yields the model with the best performance is selected. In the next iteration, it creates another set of N−1 models with two input features: the one selected in the previous iteration and another of the N−1 remaining features. Again, the combination of features that gives the best performance is selected. The script stops when it reaches the number of desired features. One improvement we made to this script was including k-fold cross-validation in the model evaluation process at each iteration. This ensured that the good or bad performance of one model was not produced by chance because of a single favorable train/test split.

**Figure 6:**
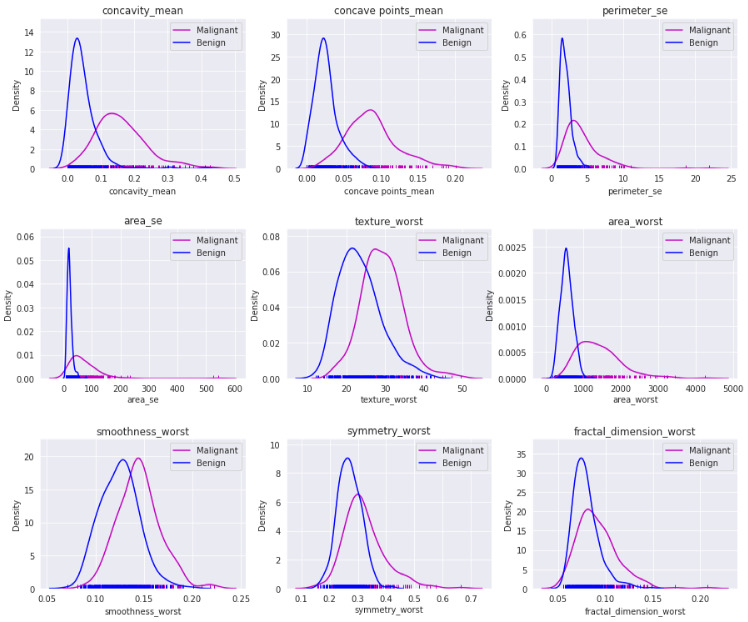
Distribution of malignant and benign cells for reduced features for WBCD.

The result provided nine attributes, concavity-mean, concave points-mean, perimeter-se, area-se, texture-worst, area-worst, smoothness-worst, symmetry-worst, and fractal-dimension-worst, as shown in Figure 6. The total number of subsets evaluated was 955, and the best subset figure of merit was 96.8%.

### 3.2. Breast Cancer Tumor Classification

The following classification algorithms were evaluated for the task of breast cancer tumor classification, and hyper-parameter tuning was performed for classifiers using “GridSearchCv”, which performs exhaustive searching over specified parameter values for an estimator. GridSearchCV tries all the combinations of the values passed in the dictionary and evaluates the model for each combination using the Cross-Validation method. From the exhaustive set of accuracies thus obtained, the best one is chosen.

#### 3.2.1. NaïVe Bayes Classification

The naïve Bayes model is very effective for large datasets because of its simplicity. It works on the probability basis p(c | x), where p(c | x) is the posterior probability of the class (c) and predictor (x).

#### 3.2.2. Support Vector Machine (SVM)

We performed hyper-parameter tuning for SVM with GridSearchCv. SVM-CV performance was then compared with default SVM performance. Both showed the same accuracy, precision, and recall score. The parameters values showing the best performances were *C* = 1 and degree = 1, where *C* is a SVM cost function used for SVM optimization and degrees is a value of polynomial used to find the hyper-plane to split the data. The default setting for SVM is *C* = 1, degree = 3.

#### 3.2.3. Decision Tree

Decision trees use multiple algorithms to decide to split a node into two or more sub-nodes. The creation of sub-nodes increases the homogeneity of resultant sub-nodes. In other words, it can be said that the purity of the node increases with respect to the target variable. The decision tree splits the nodes on all available variables and then selects the split which results in the most homogeneous sub-nodes.

#### 3.2.4. K-Nearest Neighbors (KNN)

KNN is non-parametric method, as it does not consider the dimensionality of dataset for diagnosis because it relies upon nearest training data points. The “GridSearchCv” was used to figure out the total number of neighbors for the KNN training needed to achieve superior performance.

#### 3.2.5. The Random Decision Forest Method

A random forest is considered as a highly accurate and robust method because of the number of decision trees participating in the process. It tries to build k different decision trees by picking a random subset *S* of training samples. It generates fully Iterative Dichotomiser 3 (ID3) trees with no pruning. It makes a final prediction based on the mean of each prediction, and it tends to be robust to overfitting, mainly because it takes the average of all the predictions, which cancels out biases.

#### 3.2.6. Simple Logistic Regression

Logistic regression is a statistical method for evaluating a dataset in which a result is calculated by one or more independent variables. It is a supervised learning technique similar to linear regression.

#### 3.2.7. Stochastic Gradient Descent Learning for Support Vector Machine

In stochastic gradient decent, in each interaction only a few samples are selected randomly instead of the entire dataset. The samples are shuffled at random and chosen to perform the interaction.

### 3.3. Ensemble Classification

Ensemble learning strategically brings together several machine learning models for achieving better performance. There are three different types of ensemble techniques: bagging based ensemble learning, boosting based ensemble learning, and voting-based ensemble learning. In our work, we used voting-based ensemble learning.

Voting-based ensemble learning is one of the basic or straightforward ensemble learning techniques in which diagnoses from multiple models are combined with either hard or soft voting.

#### 3.3.1. The Majority-Based Voting Mechanism (Hard Voting)

In hard voting, we assign or predict the final class label as the class label that the classification models has most often predicted. Hard voting is the simplest case of majority voting. In majority voting, the class label *y* is predicted via majority (plurality) vote the classifiers *C*:

(1) $$y\;=\;mode\;\left\{\;C_{1}\left(x\right),\;C_{2}\left(x\right),\;..,\;C_{n}\left(x\right)\right\}$$

#### 3.3.2. The Probability-Based Voting Mechanism (Soft Voting)

In soft voting, we predict the class labels based on the predicted probabilities *p* for the classifiers [23]. Soft voting attains the best results by averaging out the probabilities calculated by individual algorithms. Soft voting predicts the label as:

(2) $$\hat{y}=\underset{i}{argmax}\sum_{j=1}^{m}w_{j}p_{ij}$$

where $w_{j}$ is the weight that is assigned to the j^th^ classifier and $p_{ij}$ is the predicted membership probability of the i^th^ classifier for class label *j*.

## 4. Experimentation and Discussion

### 4.1. The Wisconsin Breast Cancer Dataset (WBCD)

This public dataset [2] is based on microscopic examination of aspiration tests using fine needles on breast masses. The breast mass attribute is determined from a digital fine-needle aspirate (FNA) scan. Breast mass FNA is an important way of assessing malignancy. The WBCD was created by Dr. William H. Wolberg at the University of Wisconsin-Madison Hospital. There are 569 instances in this database, consisting of two cases: 357 benign instances and 212 malignant ones. These 569 instances are of human breast tissue from the FNA and were clinically evaluated based on 32 characteristics. All attributes can be considered as symptoms of a patient’s breast cancer. Finally, 70:30 training:testing split was used for evaluation.

### 4.2. Results and Discussion

As can be seen in Table 1, results without pre-processing are unreliable and inaccurate. Some classifiers—support vector machine, naïve Bayes, etc.—did not perform well and produced low precision and recall scores.

After feature selection, we compared the performances of different machine learning classification methods for breast tumor classification. To find out best parameters, hyper-parameter tuning using GridSearchCv was used, and the performance of each classifier was improved after that. As shown in Table 2, logistic regression outperformed the other classifiers with an accuracy of 97.49% and high precision and recall of 97.89% and 95.21%, respectively.

Table 3 shows that probability-based soft voting mechanism performed better than majority-based (hard voting) voting, because soft voting uses more information by using individual classifiers’ uncertainties in the final diagnosis.

Results after applying wrapper feature selection methods:

The nine attributes: concavity-mean, concave points-mean, perimeter-se, area-se, texture-worst, area-worst, smoothness-worst, symmetry-worst, and fractal-dimension-worst were provided by a wrapper feature selection method. Performance results of machine learning classifiers with reduced numbers of features from the initial set are shown in Table 4. Kernel density estimation (KDE) plots were used to check the distribution of malignant and benign cases for selected features. The visualization of the above-mentioned features is shown in Figure 6

After this, we also analyzed the performance of this reduced set of features from the wrapper method when using ensemble voting. Table 5 shows that the probability-based soft voting mechanism performed better than majority-based (hard voting) voting, because soft voting gets more information by using individual classifiers’ uncertainties in the final diagnosis.

Comparing both methods for feature selection, it can be concluded that the performances of machine learning classifiers were improved at the individual level by using a wrapper method. As can be seen in Table 4, simple logistic regression learning provided 98.10% accuracy, random decision forest 96.70%, stochastic descent learning 97.40%, decision tree 96.83%, and naïve Bayes 92.80%. However, from the evaluation results of ensemble voting, there was only a small improvement for hard voting.

## 5. Comparison with Existing Work

Table 6, shows a comparison with existing work for breast cancer diagnosis using ensemble techniques. Nguyen et al. [3] analyzed the performances of different supervised and unsupervised breast cancer classification models on the WBCD dataset. They analyzed the performance of an ensemble voting method for breast cancer detection. They applied principal component analysis for feature analysis and reported an accuracy of 98.00%. Compared to this approach, our proposed feature selection and ensemble method classification shows an improvement of 1.00%.

Rodrigues et al. [11] achieved a performance of 97.80% on the WBCD dataset using a Bayesian network; however, they evaluated performance only using a machine learning classification algorithm and did not analyze the significance of important features needed for better performance. They have evaluated the performances of two different classification algorithms, i.e., a Bayesian network and a decision tree. The Bayesian network performed better than the decision tree.

To compare the performances of different classification models, Ahmed et al. [4] used data from the Iranian Center for Breast Cancer dataset and explored the risk factors for predicting breast cancer. There are some limitations in this study, as many cases were lost in the follow-up and the records with missing values were omitted. Some important variables, such as S-phase fraction and DNA index, were not included in the study because of their unavailability, which may have decreased the performances of the models.

Shravya et al. [17] used three well known classification algorithms for the detection of breast cancer. They used logistic regression, a support vector machine, and k-nearest neighbors. The SVM outperformed the other two classifiers and showed better performance with 92.70% accuracy. It is noted that there is a lot of room for improvement when the ensemble method classification is used instead of using individual classification algorithms. Darzi et al. [20] addressed feature selection for breast cancer diagnosis. Their process contains a wrapper approach based on a genetic algorithm (GA) and case-based reasoning (CBR), and reported an accuracy of 97.37% on WBCD. As compared to this approach, our proposed feature selection and ensemble method classification show an improvement of 2.00%.

Bharat et al. [12] achieved a performance of 97.3% on the WBCD dataset using naïve Bayes; however, they evaluated the performance only using a machine learning classification algorithm and did not analyze the significance of important features needed for better performance. They evaluated the performances of three different classification algorithms, i.e., naïve Bayes, the J48 network, and the RBF network. Naïve Bayes performed better than the other two. Assiri et al. [19] proposed an ensemble classifier based on a majority voting mechanism. The performances of different state-of-the-art ML classification algorithms were evaluated for the WBCD dataset, achieving an accuracy of 99.42%. However, they did not evaluate different feature selection algorithms that could help them to determine the smallest subset of features that can assist in accurate classification of breast cancer as either benign or malignant.

Lucas et al. [11] used Bayesian network and decision tree machine learning classifiers. The Bayesian network gave the best accuracy of 97.80% on WBCD. As compared to this approach, our proposed feature selection and ensemble method classification showed an improvement of 2.00%.

## 6. Conclusions and Future Work

Early detection of breast cancer is important, as it is one of the leading causes of death among women, so its detection at early stages is very important. Early breast cancer tumor detection can be improved with the help of modern machine learning classifiers. In medical research, the false positive and false negative examples have great significance, but most existing work has evaluated performance based only accuracy evaluation measure. Therefore, we focused not only on accuracy but also evaluated performance based on precision and recall. In this work, feature selection and dimensionality reduction were performed using principal component analysis and by analyzing the correlations among different sets of features and their variance. The performances of different machine learning algorithms, including logistic regression, support vector machine, naïve Bayes, k-nearest neighbor, random forest, decision tree, and stochastic gradient decent learning, were evaluated. We reported the performances of different classifiers using different performance measures, including accuracy, precision, and recall. A voting ensemble method was used to improve the performances of the classifiers. The three best classifiers were then used for final classification using a voting ensemble method. We used hard voting (majority-based voting) and soft voting (probability-based voting) for ensemble classification. The average-probability-based voting (soft voting) showed better results as compared to hard voting. For big datasets, how these machine learning classifiers algorithms behave is one of the future scopes of this project. This work could be enhanced through the use of deep learning techniques for classification and identification of particular stage s of breast cancer.

## Figures and Tables

**Table 1:** Breast cancer diagnosis without data pre-processing.

Classification Algorithms	Accuracy (%)	Precision	Recall	F-Measures	F2-Measures
Naive Bayes Classification	84.50%	0.70%	0.57%	0.62%	0.59%
Simple Logistic Regression	87.94%	0.88%	0.87%	0.87%	0.87%
Random Decision Forest Method	99.47%	0.99%	0.99%	0.98%	0.99%
Support Vector Machine	62.00%	0.62%	0.40%	0.48%	0.43%
K-Nearest Neighbor Classification	90.00%	0.89%	0.80%	0.84%	0.81%
Decision Tree	88.00%	0.88%	0.86%	0.86%	0.86%
Stochastic Gradient Decent Learning	90.30%	0.83%	0.88%	0.85%	0.86%

**Table 2:** Comparison of different classification methods on WBCD after feature scaling, and hyper-parameter tuning of features using PCA and correlation analysis.

Classification Algorithms	Accuracy	Precision	Recall	F-Measures	F2-Measures
Simple Logistic Regression Learning	97.49%	97.89%	95.21%	96.53%	95.73%
K-Nearest Neighbor Classification	97.49%	98.48%	89.70%	93.88%	91.32%
Support Vector Machine	96.23%	91.88%	93.94%	92.89%	93.52%
Random Decision Forest	94.22%	93.86%	82.88%	88.02%	84.86%
Stochastic Gradient Descent Learning	92.11%	84.38%	89.20%	86.72%	88.19%
Decision Tree	90.45%	87.14%	87.00%	87.06%	87.02%
Naïve Bayes Classification	91.60%	91.90%	91.80%	91.84%	91.81%

**Table 3:** Evaluation results of ensemble voting after pre-processing, using method 1.

Voting Classifiers	Accuracy (%)	Precision (%)	Recall (%)	F-Measures (%)	F2-Measures (%)
Soft Voting	99.00	99.29	96.00	97.61	96.64
Hard Voting	97.29	96.48	95.70	96.08	95.85

**Table 4:** Comparison of the performances of different classification methods for WBCD after applying the wrapper feature selection method.

Classification Algorithms	Accuracy	Precision	Recall	F-Measures	F2-Measures
Simple Logistic Regression Learning	98.10%	98.10%	98.10%	96.90%	98.10%
K-Nearest Neighbor Classification	95.43%	95.40%	95.40%	95.40%	95.40%
Support Vector Machine	95.80%	96.00%	95.80%	95.70%	95.83%
Random Decision Forest	96.70%	96.70%	96.70%	96.60%	96.70%
Stochastic Gradient Descent Learning	97.40%	97.40%	97.40%	97.40%	97.40%
Decision Tree	96.83%	96.80%	96.80%	96.80%	96.80%
Naïve Bayes Classification	92.80%	92.80%	92.80%	92.80%	92.80%

**Table 5:** Evaluation results of ensemble voting after pre-processing by using the wrapper features subset selection method.

Voting Classifiers	Accuracy (%)	Precision (%)	Recall (%)	F-Measures (%)	F2-Measures (%)
Soft Voting	97.70	97.70	97.70	97.70	97.70
Hard Voting	97.40	97.40	97.40	97.30	97.40

**Table 6:** Comparison with the existing work for breast cancer diagnosis.

Authors	Classifiers	Accuracy (%)
Proposed Approach	Dimensionality Reduction and Ensemble based learning	99.00
Darzi et al. [20]	CBR-Genetic (case-based reasoning)	97.37
Nguyen et al. [3]	Ensemble Method	98.00
Rodrigues et al. [11]	Bayesian Network Decision Tree	97.80 92.00
Subhani et al. [17]	Logistic Regression Support Vector Machine K Nearest Neighbor	88.00 92.70 82.00
Ahmed et al. [4]	Decision tree Artificial neural network Support Vector Machine	93.60 94.70 95.70
Lucas et al. [11]	Bayesian network J48 Decision tree	97.80 96.05
Bharat et al. [12]	Decision tree C4.5 Support Vector Machine Naive Bayes K Nearest Neighbor	95.00 96.20 97.00 91.00
Assiri et al. [19]	Ensembled machine learning method	99.42

## Data Availability

Dataset is publicly available.

## Appendix A

This section contains the information about the libraries used for implementation [24].

- Sklearn.metrics: used to import confusion-matrix
- sklearn.model-selection: used to import cross-val-core (Evaluate a score by cross-validation).
- sklearn.metrics: Used to import precision-score, recall-score, f1-score, f2-score
- NumPy: It provides support for large multidimensional array objects and various tools to work with them.
- Pandas: Pandas allow importing data from various file formats. Pandas allows various data manipulation operations such as merging, reshaping, selecting, as well as data cleaning, and data wrangling features.
- Matplotlib: Matplotlib is a plotting library for the Python programming language and its numerical mathematics extension NumPy.
- Seaborn: Seaborn is a library in Python predominantly used for making statistical graphics. Seaborn is a data visualization library built on top of matplotlib and closely integrated with pandas data structures in Python.
- Scikit-learn: Scikit-learn is also known as sklearn. It is free and the most popular machine learning library for Python and used to build machine learning models.

## References

[1] Coccia M. (2019). The increasing risk of mortality in breast cancer: A socioeconomic analysis between countries. J. Soc. Adm. Sci..

[2] UCI Breast Cancer Wisconsin Dataset. https://archive.ics.uci.edu/ml/datasets/Breast+Cancer+Wisconsin+(Diagnostic).

[3] Nguyen Q.H., Do T.T., Wang Y., Heng S.S., Chen K., Ang W.H.M., Philip C.E., Singh M., Pham H.N., Nguyen B.P. Breast Cancer Prediction using Feature Selection and Ensemble Voting. Proceedings of the 2019 International Conference on System Science and Engineering (ICSSE).

[4] Ahmad L.G., Eshlaghy A., Poorebrahimi A., Ebrahimi M., Razavi A. (2013). Using three machine learning techniques for predicting breast cancer recurrence. J. Health Med. Inform..

[5] Omondiagbe D.A., Veeramani S., Sidhu A.S. Machine Learning Classification Techniques for Breast Cancer Diagnosis. Proceedings of the IOP Conference Series: Materials Science and Engineering.

[6] Yesuf S.H. (2019). Breast cancer detection using machine learning techniques. Int. J. Adv. Res. Comput. Sci..

[7] Chen X.W., Jeong J.C. Enhanced recursive feature elimination. Proceedings of the Sixth International Conference on Machine Learning and Applications (ICMLA 2007).

[8] Jamal A., Handayani A., Septiandri A., Ripmiatin E., Effendi Y. (2018). Dimensionality Reduction using PCA and K-Means Clustering for Breast Cancer Prediction. Lontar Komput. J. Ilm. Teknol. Inf..

[9] Mandal S.K. (2017). Performance analysis of data mining algorithms for breast cancer cell detection using Naïve Bayes, logistic regression and decision tree. Int. J. Eng. Comput. Sci..

[10] Kumar V., Mishra B.K., Mazzara M., Thanh D.N., Verma A. (2020). Prediction of Malignant and Benign Breast Cancer: A Data Mining Approach in Healthcare Applications. Advances in Data Science and Management.

[11] Borges L.R. (1989). Analysis of the wisconsin breast cancer dataset and machine learning for breast cancer detection. Group.

[12] Bharat A., Pooja N., Reddy R.A. Using Machine Learning algorithms for breast cancer risk prediction and diagnosis. Proceedings of the 2018 3rd International Conference on Circuits, Control, Communication and Computing (I4C).

[13] Aavula R., Bhramaramba R. (2019). XBPF: An Extensible Breast Cancer Prognosis Framework for Predicting Susceptibility, Recurrence and Survivability. Int. J. Eng. Adv. Technol..

[14] Chicco D., Rovelli C. (2019). Computational prediction of diagnosis and feature selection on mesothelioma patient health records. PLoS ONE.

[15] Wang H., Yoon S.W. Breast cancer prediction using data mining method. Proceedings of the IIE Annual Conference, Institute of Industrial and Systems Engineers (IISE).

[16] Abdolell M., Tsuruda K.M., Lightfoot C.B., Payne J.I., Caines J.S., Iles S.E. (2016). Utility of relative and absolute measures of mammographic density vs clinical risk factors in evaluating breast cancer risk at time of screening mammography. Br. J. Radiol..

[17] Shravya C., Pravalika K., Subhani S. (2019). Prediction of Breast Cancer Using Supervised Machine Learning Techniques. Int. J. Innov. Technol. Explor. Eng. (IJITEE).

[18] Williams K., Idowu P.A., Balogun J.A., Oluwaranti A.I. (2015). Breast cancer risk prediction using data mining classification techniques. Trans. Networks Commun..

[19] Assiri A.S., Nazir S., Velastin S.A. (2020). Breast tumor classification using an ensemble machine learning method. J. Imaging.

[20] Darzi M., AsgharLiaei A., Hosseini M., others (2011). Feature selection for breast cancer diagnosis: A case-based wrapper approach. Int. J. Biomed. Biol. Eng..

[21] Marrone S., Piantadosi G., Fusco R., Petrillo A., Sansone M., Sansone C. Breast segmentation using Fuzzy C-Means and anatomical priors in DCE-MRI. Proceedings of the 2016 23rd International Conference on Pattern Recognition (ICPR).

[22] Rundo L., Militello C., Tangherloni A., Russo G., Vitabile S., Gilardi M.C., Mauri G. (2018). NeXt for neuro-radiosurgery: A fully automatic approach for necrosis extraction in brain tumor MRI using an unsupervised machine learning technique. Int. J. Imaging Syst. Technol..

[23] Wang H., Yang Y., Wang H., Chen D. (2013). Soft-voting clustering ensemble. International Workshop on Multiple Classifier Systems.

[24] Raschka S. (2015). Python Machine Learning.

